# Epigenome-Wide Association of Infant Feeding and Changes in DNA Methylation from Birth to 10 Years

**DOI:** 10.3390/nu13010099

**Published:** 2020-12-30

**Authors:** Yamini Mallisetty, Nandini Mukherjee, Yu Jiang, Su Chen, Susan Ewart, S. Hasan Arshad, John W. Holloway, Hongmei Zhang, Wilfried Karmaus

**Affiliations:** 1 Division of Epidemiology, Biostatistics and Environmental Health, School of Public Health, University of Memphis, Robison Hall, Memphis, TN 38152, USA; ymllstty@memphis.edu (Y.M.); nmkhrjee@memphis.edu (N.M.); yjiang4@memphis.edu (Y.J.); hzhang6@memphis.edu (H.Z.); 2 Department of Mathematical Science, University of Memphis, Dunn Hall, Memphis, TN 38152, USA; schen4@memphis.edu; 3 College of Veterinary Medicine, Michigan State University, East Lansing, MI 48824, USA; ewarts@msu.edu; 4 NIHR Southampton Biomedical Research Centre, University Hospital Southampton NHS Foundation Trust, Southampton SO16 6YD, UK; S.H.Arshad@soton.ac.uk (S.H.A.); J.W.Holloway@soton.ac.uk (J.W.H.); 5 Clinical and Experimental Sciences, Faculty of Medicine, University of Southampton, Southampton SO17 1BJ, UK; 6 The David Hide Asthma and Allergy Research Centre, St Mary’s Hospital, Isle of Wight PO30 5TG, UK; 7 Human Development and Health, Faculty of Medicine, University of Southampton, Southampton SO16 6YD, UK

**Keywords:** epigenome-wide association study, EWAS, epigenetics, DNA methylation, infant feeding, feeding mode, breastfeeding, formula feeding, mixed feeding, delta residuals

## Abstract

Epigenetic factors have been suggested as mediators of early-life nutrition to future health. Prior studies focused on breastfeeding effects on DNA methylation (DNAm), ignoring other feeding modes. In this analysis of the Isle of Wight birth cohort, feeding modes were categorized as exclusive breastfeeding (EBF), exclusive formula feeding (EFF), and mixed feeding based on whether the respective feeding mode lasted for at least 3 months. In addition, in the past, infant feeding modes were assessed using DNAm at one time point in childhood, not changes of DNAm. In this paper, methylation differences (delta DNAm) were calculated by subtracting residual methylation values at birth from age 10 years (adjusting for cell types and season of blood collection at both ages). These deltas were estimated for all methylation sites where cytosine was followed by guanine (cytosine guanine dinucleotide (CpG) sites). Then, we performed an epigenome-wide association study contrasting EBF, EFF, and mixed feeding with delta DNAm that represents changes in methylation from birth to 10 years. A total of 87 CpGs (EBF: 27 CpGs, EFF: 48 CpGs, mixed: 12 CpGs) were identified using separate linear regression models adjusting for confounders and multiple testing. The sum of all changes in methylation from birth to age 10 years was significantly lower in the EFF group. Correspondingly, the number of CpGs with a methylation decline was 4.7% higher reflecting 13,683 CpGs. Lower methylation related to exclusive formula feeding and its adverse potential for the child’s development needs future research to reduce adverse health effects.

## 1. Introduction

Adequate nutrition throughout infancy and early childhood, especially during the first two years of life, is crucial for ensuring good health, optimal growth and development of newborns to their full potential [1,2]. Infant feeding practices include breast milk (BM) feeding and supplementary feeding (any food/liquid, including non-human milk or formula) [3]. Breast milk feeding can be distinguished into exclusive breastfeeding (BM along with vitamin/mineral supplements or medication), predominant breastfeeding (BM along with water, water-based drinks, fruit juice, vitamin/mineral supplements or medicine), and breastfeeding (BM along with any food/liquid, including non-human milk or formula) [3]. The World Health Organization (WHO) and several other organizations recommend exclusive breastfeeding for the first six months of life with the introduction of nutritionally adequate and safe complementary feeding starting from the age of six months with continued breastfeeding up to 2 years of age or beyond [4,5,6]. Despite infant formula trying to mimic the nutrition profile of human breast milk, BM stands out in terms of the colostrum/first fluid with unique immunologic components including anti-inflammatory, growth factors, and diverse gut microbiome [7,8,9]. Furthermore, the composition of human breast milk is dynamic as it changes over time within nursing sessions (foremilk and hindmilk), different stages of lactation (age of infant), and is surprisingly related to infant gender [10,11,12]. Compared to breastfed children, formula-fed children have higher risks of infectious morbidity, lower IQ scores, and are more likely to develop obesity, type 2 diabetes, and cardiovascular disease [13,14]. 

Epidemiological studies propose that epigenetics may constitute one of the potential mechanisms linking early life nutritional exposure with health and diseases in later life [15,16]. Epigenetics is the study of changes in gene activity that are mitotically heritable without changes in the DNA sequence. One of the most commonly studied epigenetic mechanisms related to early life nutrition is DNA methylation (DNAm)—the addition or removal of a methyl group most commonly at cytosine guanine dinucleotides (CpGs) [17,18]. DNAm has been proposed as a mechanism behind the long-term health effects of breastfeeding [19]. A recent systematic review suggests that findings from previous association studies on breastfeeding and DNAm are inconclusive and recommends more epigenome-wide association studies (EWASs) with proper measures of relevant covariates to be performed [20]. Our literature search identified ten association studies on breastfeeding and DNAm in humans (two candidate gene studies [21,22], two EWASs with breastfeeding as a covariate [23,24], and six EWASs with breastfeeding as an exposure variable [25,26,27,28,29,30]) (Table S1). It is important to note that these studies were highly diverse in terms of the age of assessment, tissue examined, array platform, DNAm targets and respective methods, and the categorization of breastfeeding. In addition, there is only one study that compared soy- and cow-based infant formula [31]. However, recent investigations on infant feeding and gene expression showed that infants’ current weight, height, and head circumference and expression of obesity-predisposing genes (*FTO* and *CPT1A)* are significantly higher in infants that were formula or mixed fed in comparison to exclusive breastfed [32]. Moreover, a mixed feeding mode (direct feeding at the breast, pumping and feeding, and formula feeding) was associated with a higher risk of food allergy symptoms than a single infant feeding source such as direct feeding at the breast and formula feeding [33].

Besides the limitations of prior association studies discussed in the recent systematic review [20], we identified two additional limitations in their study designs. First, most of the studies when assessing effects of breastfeeding duration on the epigenome were conducted irrespective of formula or solid feeding [25,26,27,28], which can be confounded by effects of breastfeeding. For example, infants could be mostly formula fed but still classified as breastfeeding in categorizations that have been widely investigated. To overcome this limitation, we used a more stringent categorization of infant feedings, such as exclusive breastfeeding, exclusive formula feeding and mixed feeding in our analysis. Second, past DNAm studies focused on measurement of DNAm at only one time point, e.g., 10 or 18 years [29], ignoring that the baseline of methylation can already be different at birth (before breastfeeding) and that DNAm at one point in childhood does not estimate changes that occur as a consequence of breastfeeding. Hence, only investigating the differences before and after breastfeeding, i.e., from birth to certain age, will provide appropriate assessments of epigenetic effects of infant feeding. Therefore, the objectives of this study are three-fold, (1) to examine whether DNAm changes from birth to ten years upon exposure to three infant feeding modes and if so, (2) to investigate the direction of association observed in each feeding group, and (3) to explore the effects of infant feeding on overall DNAm. Finally, we check whether the identified CpGs or genes were also reported in prior studies.

## 2. Materials and Methods

### 2.1. Study Setting and Participants

The data analyzed in this study are from the Isle of Wight Birth Cohort (IOWBC), the F1-generation (IoW F1). The IOWBC is a longitudinal cohort established to study the natural history of asthma and allergic conditions prospectively. The Isle of Wight is a small island (∼20 miles across) close to the British mainland with no industrial exposure and consists of primarily Caucasian participants (>98%). Potential study participants (n = 1536) were recruited between 1989 and 1990 by contacting parents (first generation, IoW F0) of all infants born on the IoW during this period, and approximately 95% of infants (n = 1456) were enrolled following informed consent and after exclusion of perinatal deaths, adoptions, moving, and refusals. Offspring were then followed at 1, 2, 4, 10, 18, and 26 years of age. Information about infant nutrition such as breastfeeding duration and introduction of formula and solids was gathered using questionnaires. The details on the study design, ethical approvals, enrollment, follow-up procedures, and variables assessed are described elsewhere [34,35]. Our analysis focused on the DNAm measurements at birth in DNA extracted from neonatal heel prick blood spots on Guthrie cards (n = 796), and at age 10 years (n = 330) in DNA from peripheral blood; 221 participants had DNAm data at both ages. After exclusion of missing information related to infant feeding, the analytical sample includes 201 participants. 

### 2.2. Study Variables

#### 2.2.1. Infant Feeding

Information on breastfeeding practices and total breastfeeding duration (relates to the number of weeks a mother breastfed her child regardless of the introduction of formula and/or solid food) and introduction of formula and solids were obtained through questionnaires answered by the mothers at the 1- and 2-year follow-up. For our analysis, we calculated exclusive breast feeding and exclusive formula feeding and mixed feeding duration (a combination of either of three feeding modes, breastfeeding/formula/solids) from the original feeding variables and used them as categorical variables. We used three months (13 weeks) as a cutoff point to categorize participants based on whether they were exclusively breastfed, exclusively formula fed, or a combination of any of the two feedings modes. Each feeding mode is determined as follows.

- Exclusive Breastfeeding (EBF) group: child was only breastfed for at least 13 weeks of age before formula and/or solid foods were introduced.
- Exclusive Formula Feeding (EFF) group: child was only formula fed (or cow’s milk) for at least 13 weeks of age (no breastfeeding) before solids were introduced.
- Mixed feeding group: child was fed a mixture of breastmilk, formula, and solids before 13 weeks of age.

#### 2.2.2. DNA Extraction and Profiling

DNA was extracted from the peripheral blood samples at age 10 (n = 330) using a standard salting-out procedure [36] and from dried heel blood samples (Guthrie cards) collected after the birth (n = 796) using a method based on the procedure described by Beyan et al. [37]. Approximately one microgram of DNA was bisulfite-treated for cytosine to thymine conversion using the EZ 96-DNA methylation kit (Zymo Research, Irvine, CA, USA) following the manufacturer’s standard protocol. Epigenome-wide DNAm was measured at birth and age 10 using Illumina BeadChip arrays (Illumina, Inc., San Diego, CA, USA). DNAm from Guthrie cards after birth were assessed using the Infinium Methylation EPIC BeadChip arrays measuring >850K CpGs. At age 10 years, both Infinium HumanMethylation450 BeadChip and EPIC BeadChip arrays were used and only CpG sites common between the two platforms were included in the analyses.

Methylation data were quantile normalized using the minfi R package and batch corrected using ComBat in R [38]. Probes not reaching a detection *p*-value of 10^−16^ in at least 95% of samples were excluded. To reduce the possible influence of probe single nucleotide polymorphisms (SNPs), CpG sites with probe SNPs within 10 base pairs and with minor allele frequency > 7% in the Caucasian population were further excluded. These steps resulted in 551,710 CpGs from 796 participants at birth, and 349,455 CpGs from 330 participants at age 10 years. Autosomal probes were then selected and converted to beta values, which represents the ratio of methylated (M) over the sum of methylated and unmethylated (U) probes (β = M/[c + M + U]), where c is used as a constant to prevent zero in the denominator. Finally, a total of 292,366 CpGs (present both at birth and age 10) were included in the statistical analyses.

#### 2.2.3. Covariates

Variables known to potentially confound the association between duration of infant feeding and offspring DNAm levels were selected as covariates. These include categorical variables such as maternal smoking, birth order, socioeconomic status (SES), mode of delivery, and maternal asthma, and continuous variables such as birth weight and maternal age. Maternal age at delivery (in years) was calculated using child’s and maternal date of birth. Information about maternal smoking during pregnancy (question on active smoking) and maternal asthma (obtained from the questions of whether the mother suffered from asthma or wheezing attacks) were reported by the mothers at birth. Mode of delivery (recorded as Cesarean delivery, yes vs. no) and birthweight in kilograms were ascertained from the birth records. The birth order of the index child in the family (1st/2nd/3rd or more) was gathered by questionnaire. A composite family socioeconomic status (SES)-cluster variable that included a combination of parental occupation (reported at birth), number of children in a child’s bedroom (collected at age 4 years), and family income (at age 10) was used in our analyses. This composite variable has been described elsewhere [39].

In addition to covariates primarily related to breastfeeding, we also adjusted for variables that may confound effects of DNAm. These include infant’s sex [40], estimated cell-type composition of blood samples [41,42] and season of blood collection [43,44] at birth and age 10 years. Child’s sex was used to control the gender effects on the DNAm. We estimated proportion of cell types (CD8T, CD4T, natural killer cells, B Cells, monocytes, granulocytes) at each age (birth and 10 years) using the estimateCellCounts function from the minfi package [45] with an adult reference panel [42] following the Houseman approach [46]. Since Guthrie cards were collected between Day 3 and 5 after delivery, there was no need to additionally adjust the DNAm for nucleated red blood cells (nRBCs), as on Day 3 to Day 5 after birth nRBCs are no longer seen in the blood circulation of the newborn [47,48,49].

Season of blood collection at birth (obtained from the date of birth) and 10 years (obtained from the date of interview at 10 years of follow up) were regrouped as December–February (winter) March–May (spring), June–August (summer), and September–November (Fall) and used winter as reference [43]. 

### 2.3. Statistical Analysis

#### 2.3.1. Comparison of the Analyzed Sample with the Complete Cohort

To evaluate whether the analyzed sample (subjects with DNAm data at birth and 10 years) differed from the study cohort, we compared the sample with the population using one-sample proportion tests (for categorical variables) and one-sample t-tests (for continuous variables). 

#### 2.3.2. Estimation of Differences of DNAm from Birth to Age 10 Years

The analyses were restricted to paired data, i.e., CpGs and participants present both at birth and 10 years (292,366 CpGs and 201 participants). In the first step, to achieve normal distributions of DNAm measurements at birth and 10 years, we logit-transformed beta values to M values (using log2 (β value/(1-β value)). We then regressed M values on cell-type proportions and season of blood collection for each age (birth and 10 years) separately. This provides the residuals of DNAm (residDNAm) at birth and 10 years representing DNAm after correcting for cell types and season of blood collection. To determine differences in DNAm between birth and 10 years (delta or ‘Δ’ or delta-residuals (ΔDNAm)), the difference between the residuals at birth and 10 years was calculated (ΔDNAm = residDNAm at age 10–residDNAm at Birth). The delta-residuals (ΔDNAm) were treated as the outcome for further analysis.

#### 2.3.3. Epigenome-Wide Discovery of Statistically Important CpGs Related to Infant Feeding

In the second step, we used delta-residuals, the changes in DNAm from birth to age 10 years, as an outcome to perform three separate EWASs for each infant feeding variable (EBF, EFF, and mixed) with two categories as the exposure by linear regression models. We screened all CpGs using the *ttScreening* R package, which was applied on delta-residuals to identify CpGs associated with each infant feeding variables separately using the following conceptual model:

ΔDNAm(CpGi) = α + (β1 × feeding variable)

where ΔDNAm(CpGi) denotes changes in DNAm at methylation site i and feeding variable represents one of the independent variables: EBF group, EFF group, and mixed group. This method employs training and testing data in robust linear regressions with surrogate variables in the regressions to adjust for unexplained variation in the data. The approach implemented in ttScreening was shown to perform better in controlling types I and II errors than the method controlling the false discovery rate (FDR) and the Bonferroni method [50]. CpGs that were not associated with infant feeding were excluded. 

#### 2.3.4. Linear Regression Adjusting for Confounder of the Discovered CpG Sites

To adjust for potential confounding, linear regression models were used for those CpGs that passed the screening with delta-residual at each CpG site as a dependent variable. All potential confounders (infant’s sex, cell compositions and season of blood collection at birth and age 10 years, maternal age at delivery, maternal smoking during pregnancy, mode of delivery, birthweight, birth order, and family socioeconomic status) were included in the model. Next, we compared the direction of significant regression coefficients across all three infant feeding modes using bar and volcano plots.

Additionally, we tested whether the overall global DNAm of 292,366 CpGs changed from birth to age 10 years. To this end, we first calculated the mean of the delta-residuals for each of the 201 subjects across all the 292,366 CpGs (paired data at birth and ten years). We then fitted a linear regression model using the means of all individual ΔDNAm as outcomes and the three infant feeding modes as exposures controlling for confounders (infant’s sex, cell compositions and season of blood collection at birth and age 10 years, maternal age at delivery, maternal smoking during pregnancy, mode of delivery, birthweight, birth order, and family socioeconomic status). Second, to investigate whether the overall DNAm was affected by infant feeding, we repeated the above equation with the average DNAm over all CpGs as outcome. Then, to test whether overall DNAm changes is due to minimal changes of all CpGs or higher changes in a limited proportion, we tested the proportion with positive (increase of DNAm) and negative changes (decrease in DNAm). To perform this, we first calculated the upper limit Q1 (lower quartile) and the lower limit Q3 (upper quartile) of all 292,366 CpGs for each of the 201 participants. Then, we determined the population medians of these Q1s and Q3s over all 201 children. For each child, we computed the percentage of CpGs below the median of the population-based Q1 (representing negative changes) and the corresponding percentage of CpGs above the median of the population-based Q3 (representing positive changes). Finally, we conducted two separate linear regression models using the percentages of CpGs (for 292,366 CpGs and 201 participants) that are below the population-based Q1 and the percentage of CpG that are above the Q3. Again, in these models, the three infant feeding modes were the exposures and we adjusted for confounders.

**Figure 1:**
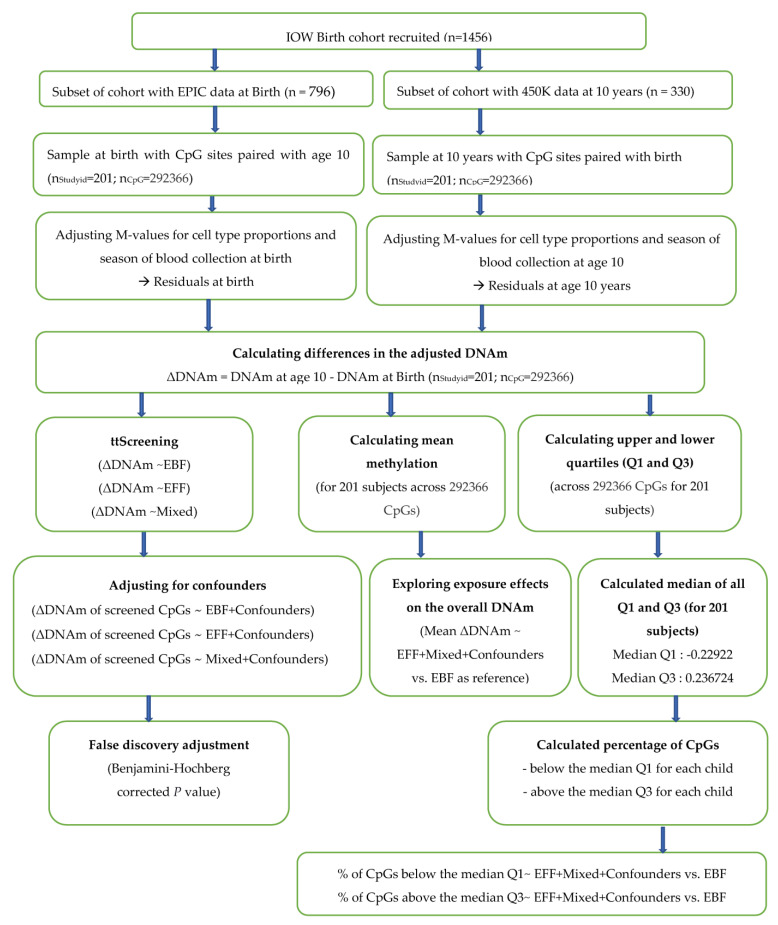
Flow chart of statistical analyses. nStudyid and nCpG represent the number of study participants and CpGs considered in the analysis, DNAm (DNA methylation), and Residual-DNA methylation after taking out the effects of cell-type proportions and season of blood collection at both time points. ttScreening—training and testing screening method, EBF—exclusively breastfed for at least 13 weeks, EFF—exclusively formula fed for at least 13 weeks, Mixed—a mixture of breastmilk with formula or solids before 13 weeks, ΔDNAm M-values—delta DNA methylation (calculated using M-values) that represents change in DNA methylation from birth to 10 years after adjusting for the cell-type proportions and season of blood collections at both time points.

For all outcomes of the linear regression analyses, we checked the multi-variate normal distribution. The flow of the statistical analysis is shown in Figure 1. In all these analyses, multiple testing was adjusted by controlling a false discovery rate (FDR) of 0.05. Analyses were conducted using R package (version 3.6.1; Vienna, Austria) and SAS (version 9.4; SAS Institute, Cary, NC, USA).

### 2.4. Agreement of CpGs or Genes from Our Results with Prior Findings

In addition to statistical analysis, we performed a follow-up investigation of our findings to examine if we could find agreement between CpGs or genes from our analysis with those reported previously. Reports that used breastfeeding as a confounder, e.g., analyzed in tumor samples or studies that did not provide CpG sites information were excluded. The final list includes a total of 4308 CpGs, i.e., 12 CpGs from the study of Odintsova et al. [30], 13 CpGs from the study of Sherwood et al. [27,32], 7 CpG from Hartwig et al. [28], and 4276 CpGs from Naumova et al. [29]. We then used this list to look for the overlap of CpGs or their mapped genes with our findings. 

### 2.5. Selective Screening of Associations with Continuous Health Outcomes

The scope of this paper does not allow for a systematic investigation of long-term health effects related to potential stable CpGs. However, within our study focusing on allergies and related factors, we tested the role of more stable CpGs for three outcomes with a continuous scale: one lung function test, serum IgE levels (a marker of allergy), and body mass index (BMI) at age 18. Measurements of these markers have been reported in prior papers [51,52,53].

Since health effects later in life require some stability of the DNAm changes, before linking Δ residuals of the DNAm to these three health outcomes, we tested how many of the discovered CpGs can be characterized as showing little dynamic in their methylation levels and thus could be classified as informative biomarkers in contrast to random effects. Using further information on DNAm at 18 years [54], we used mixed linear regression to check the temporal variability of the methylation of discovered CpGs between 10 and 18 years. To err on the safe side, we considered methylation changes between 10 and 18 years as dynamic, if there was a statistical indication of possible temporal changes with a *p*-value of 0.1 and no adjustment for multiple testing.

## 3. Results

### 3.1. Description of Study Participants

Among the 1456 enrolled children in the original cohort, 1360 were followed to age 10, of which 796 and 330 participants had DNA methylation available at birth and 10 years, respectively. For our analyses, we included 201 participants with methylation data for both ages and with non-missing information for infant feeding variables. The descriptive statistics comparing analyzed samples with the whole cohort are summarized in Table 1. The mean duration of total breastfeeding, the introduction of formula, and the introduction of solids were 14 weeks (±4.3), 8.9 weeks (±10.9), and 14.3 weeks (±4.6), respectively. The proportions of infants exclusively breastfed and formula fed for more than three months are 18.2% and 8.9%, respectively. Most infants (72.8%) received formula or solid foods before three months.

The subsamples included in the study represented the complete cohort with respect to all exposure and confounding variables included in the analysis, except for season of heel prick blood collection at birth, maternal age at delivery, and age at the introduction of solids. Significant differences were observed in the proportion of children born during the spring (20% vs. 14%) and summer (15% vs. 18%) for season of blood collection at birth. The average maternal age (29.2 vs. 29.6) and age of solid food introduction in weeks (14.3 vs. 15.2) were slightly higher in the analytical sample than the whole cohort. All these variables were controlled for in our analyses as potential confounders. 

### 3.2. EWAS of Infant Feeding Duration and Changes in DNA Methylation

**Figure 2:**
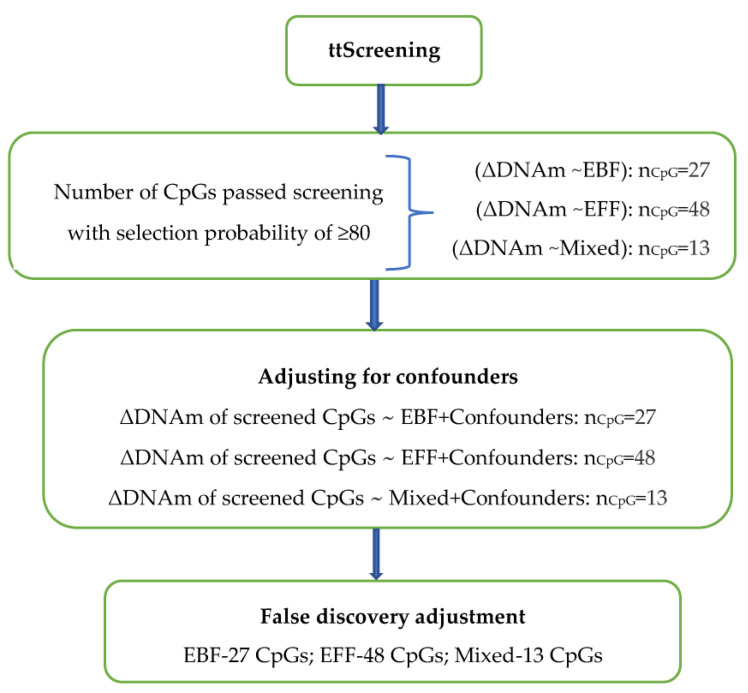
Number of CpGs associated with each infant feeding variable at each stage of analysis. ttScreening—training-testing screening method, ΔDNAm—delta DNA methylation (calculated using M-values) that represents change in DNA methylation from birth to 10 years after adjusting for the cell-type proportions and season of blood collections at both time points, EBF—exclusively breastfed for at least 13 weeks, EFF—exclusively formula-fed for at least 13 weeks, Mixed—a mixture of breastmilk with formula or solids before 13 weeks. nCpG indicates the number of CpGs that are significant with a *p*-value of <0.05.

The training-testing method (*ttScreening*) analyzing 292,366 delta-residuals detected 87 informative CpGs across all the three feeding variables (27 CpGs for EBF, 48 CpGs for EFF, and 12 CpGs for mixed feeding) (Figure 2). The delta-residuals of these CpGs were further analyzed using linear regression models by controlling for confounders. After adjusting for multiple testing (FDR = 0.05), all CpGs remained significantly associated with three infant feeding variables (Table 2). 

**Figure 3:**
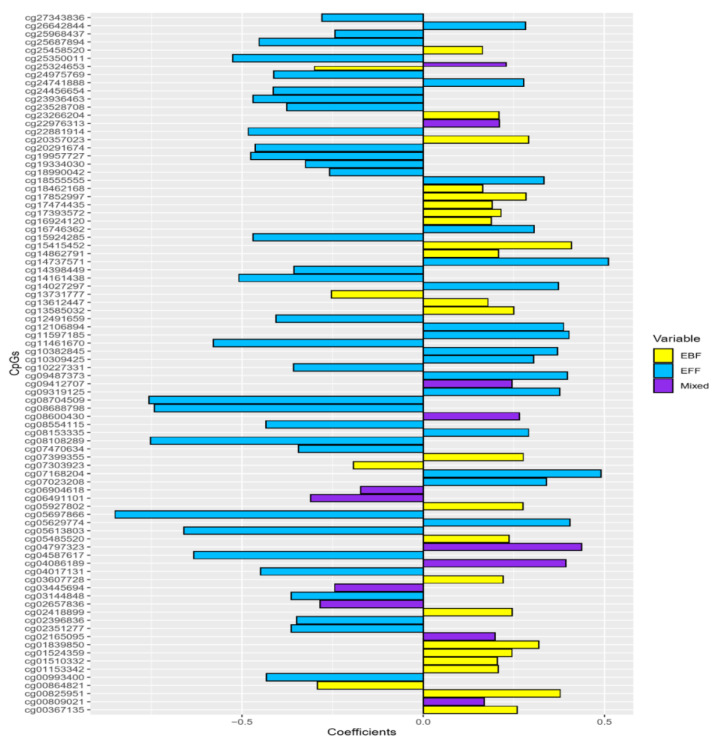
Bar plots of the regression coefficients by type of infant feeding mode. A negative coefficient represents methylation levels that are higher at birth and decreased at age 10 after exposure to different modes of feeding. A positive coefficient between ΔDNA methylation and feeding modes indicates that infant feeding is related to an increase in DNAm from birth to age 10.

**Figure 4:**
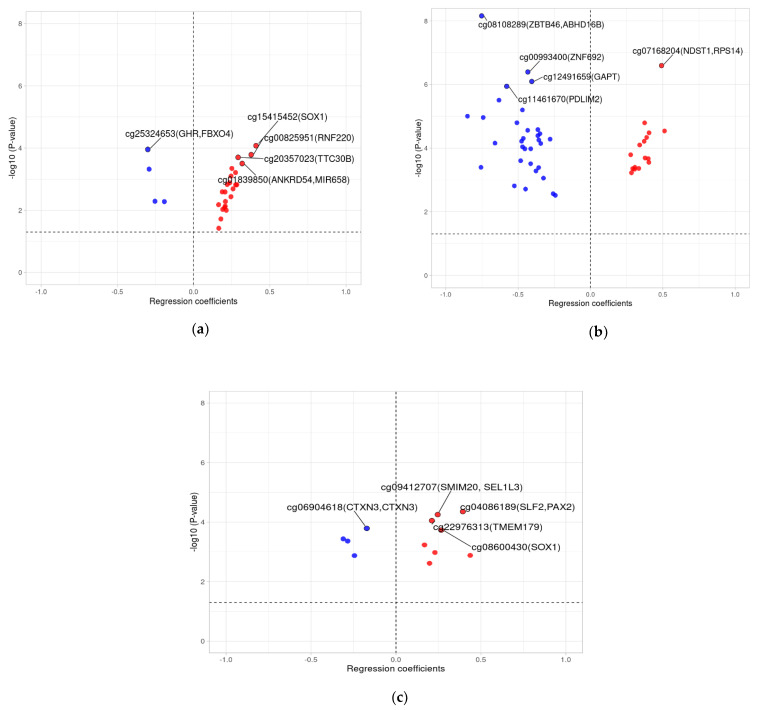
Volcano plots of −log10 (*p*-value) against regression coefficients of each infant feeding mode after adjusting for the confounders. The lower part of the volcano plot is not shown. The x-axis represents change of DNAm from birth to 10 years and the y-axis is −log10 transformed raw *p*-values (−log10p). The red-color dots denote positive regression coefficients and are towards the right side, and the blue-color dots represent negative coefficients are towards the left side of the plots. The horizontal dashed line indicates cutoffs for significance of raw *p* < 0.05, vertical dashed lines indicate regression coefficient cutoff of zero. (**a**) Volcano plot showing a total of 27 CpG sites methylated in the exclusive breastfeeding group with *p* < 0.05. (**b**) Volcano plot showing a total of 48 CpG sites methylated in the exclusive formula feeding group with *p* < 0.05. (**c**) Volcano plot showing a total of 12 CpG sites methylated in mixed feeding group with *p* < 0.05.

Inspecting the direction of regression coefficients in each infant feeding mode using bar and volcano plots shows that more CpGs related to EFF have negative regression coefficients (representing decreased methylation from birth to 10 years) than EBF and mixed feeding (Figure 3 and Figure 4). 

A linear regression model suggests that globally, over all 292,366 CpGs, the average DNAm declined more in exclusive formula-feed infants compared breastfed infants (Table 3). The mixed feeding mode showed no statistically significant association with the overall changes in DNAm between birth and 10 years of age. 

In addition, the percentage of CpGs with a more pronounced reduction in DNAm (ΔDNAm in the lower quartile of the distribution) was 4.7% (reflecting 13, 683 CpGs) higher in exclusive formula-feed compared to breast-fed infants controlling for confounders (Table 4). No associations were found for the mixed infant feeding mode and for the percentages of ΔDNAm increases over all CpGs. The average DNAm changes and the percentages of DNAm in the lower and upper quartile did not deviate from a multi-variate normal distribution. 

Interestingly the 87 CpGs discovered for exclusive breastfeeding (EBF), exclusive formula feeding (EFF), and mixed feeding were also statistically significantly differently distributed on locations on the genes (Table 5). The 27 CpGs related to EBF were more found in the transcription start site (48%), whereas the 48 CpGs related to EFF and the 12 CpGs linked to mixed feeding were found more in the body of the gene (35.4% and 50%, respectively).

According to gene annotation, the 87 CpG sites having significantly different delta-residuals and associated with three feeding variables represent 117 genes. Of these 87 CpGs, we found only one common CpG (cg25324653; *GHR*, *FBXO4*) that was associated with both exclusive breastfeeding and mixed feeding (Table 2). We also discovered genes with more than one CpG linked to different feeding modes, i.e., *SOX1* for cg15415452 (EBF; β = 0.409; P = 1.30 × 10^−3^) and cg08600430 (Mixed; β = 0.266; P = 4.00 × 10^−4^) and *ANGPT2/MCPH1* for cg04017131 (EFF; β = −0.449; P = 2.00 × 10^−3^) and cg25350011 (EFF; β = −0.526; P = 1.60 × 10^−3^).

### 3.3. Agreement of CpGs and Genes Related to Infant Feeding from Prior Studies

We investigated the agreement of CpGs or genes that we identified with those reported in prior studies. Of the 4308 reported CpGs and their mapped genes (Table S2), we found no overlap in specific CpGs between our analysis with prior studies. However, we identified seven genes (*RNF220, SFMBT2, LGI1, SUDS3,* and *CRYL1* associated with EBF; *RBFOX3* and *DDAH2* associated with EFF) from our study that were also reported previously (Table S3). 

### 3.4. Associations with Continuous Health Outcomes

**Figure 5:**
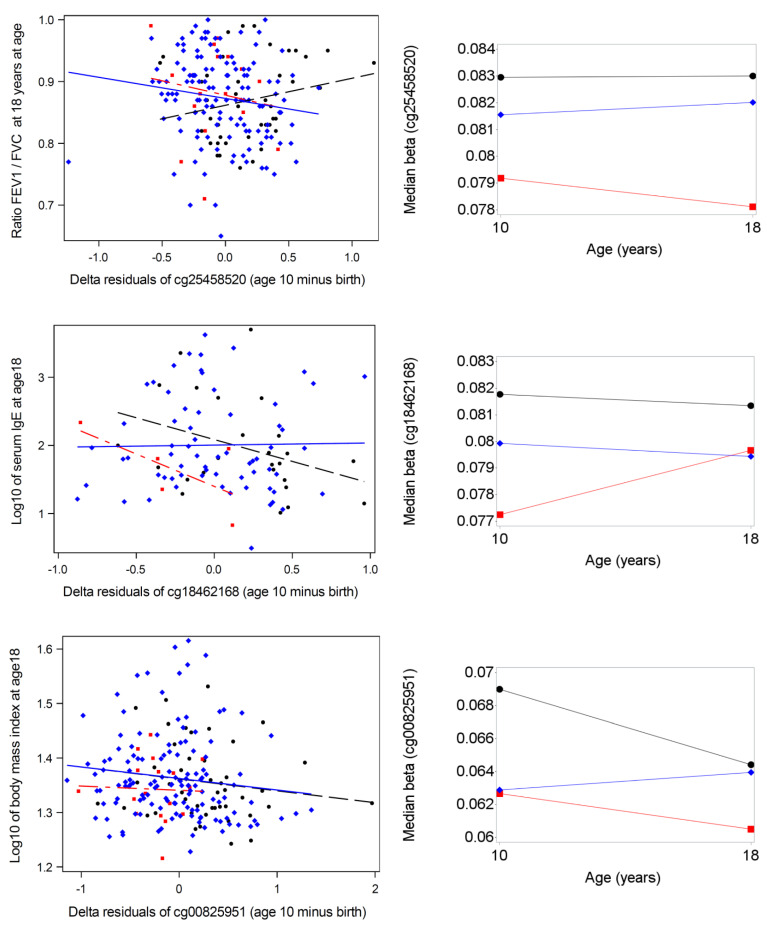
Scattergrams with regression lines for three different continuous health outcomes at age 18 years with delta residuals of three different CpGs and (left site) and medians of three CpGs at ages 10 and 18 years (N = 298) for three infant feeding groups: exclusive breastfeeding for 13 weeks—black, exclusive formula feeding for 13 weeks—red, mixed feeding mode—blue (right side). The ratio of forced expiratory flow in 1 s and forced vital capacity (FEV1/FVC) was based on lung function test at age 18, immunoglobulin E (IgE) was measured in serum in IU/mL, body mass index is defined as kg/m^2^.

Testing the temporal variability between 10 and 18 years showed that 43 of the 86 CpG showed variability, but 43 CpG were more stable between 10 and 18 years, which nevertheless is a risky period with a number of changes related to puberty [55]. For the ratio of forced expiratory flow in 1 s and forced vital capacity (FEV1/FVC) Figure 5 (left site) shows that the increasing methylation of CpG site cg25458520 (MAPK13 gene) is related to an increase in FEV1/FVC if the child was exclusively breastfed (EBF) for 13 weeks, but to a reduction if the child was formula-fed (EFF) or received mixed feeding. However, the negative association was not as strong among formula-fed children, since the methylation levels were smaller. For immunoglobulin E (IgE, log10-scale), the figure shows a protective effect of both exclusive breastfeeding and formula feeding. Again, in EFF children, the protective effect did not reach the potential of EBF, since methylation levels of cg18462168 (CYFIP1 gene) in the EFF group were smaller. In the third example (Figure 5), higher methylation levels in all infant feeding groups were related to a smaller body mass index (BMI, log10 level). However, EFF children had smaller methylation and thus could not reach the potential of higher BMI reduction. More stable median levels in the three infant feeding groups are displayed on the right side of Figure 5. The Y-axis shows the proportion of methylation (ranging from 0 to 1). The CpGs sited were selected by excluding high variability in mixed linear models of repeated DNAm levels at ages 10 and 18 years.

## 4. Discussion

Infant nutrition is essential for growth, development, and overall future health. Although various infant feeding modes exist, most of the epigenetic studies focus only on the crude effect of breastfeeding on DNAm and at a single age in childhood. To our knowledge, this is the first EWAS to investigate the association between exclusive and mixed infant feeding modes and epigenome-wide change in DNAm from birth to 10 years. In the Isle of Wight Birth Cohort, we found that exclusive breastfeeding, exclusive formula feeding, and mixed feeding were significantly associated with DNAm at 27, 48, and 13 CpGs, respectively. The direction of DNAm changes (ΔDNAm) was surprising. Among the 87 identified CpG, we found a clear tendency for reduction of DNAm from birth to age 10 years for formula-fed infants. This motivated us to test overall changes. Considering the mean of changes of all 292,366 CpGs, formula-fed infants had a statistically significant lower average change, suggesting a reduction of their methylation levels at 10 years. Testing how many CpGs show a stronger reduction (lower quartile of the population-based distribution over all 292,366 CpGs) revealed that a statistically significant proportion of 4.7% of all CpGs (corresponding to 13,683 CpGs) are lowered from birth to age 10 years in formula-fed compared to breastfed children. This suggests that children who were given formula exclusively for at least three months had significantly lower methylation (both for the identified infant-feed mode associated CpGs and global DNAm changes) than children who were exclusively breastfed. In addition to the different direction of the methylation changes of different feeding modes, we also found that CpGs related to EFF and mixed feeding were more frequently found in the body of the gene, where CpGs related to EBF were often located in the transcription start site of the gene. It is possible that both the different direction of methylation changes and the location of the affected CpGs on the gene are associated. For instance, decreased in methylation and CpGs in the body of the gene may indicate a different genetic ‘programming’ [56]. However, these aspects cannot be covered in the scope of this paper but needs further analyses.

We considered the possible effects of most practiced infant feeding modes on DNAm changes by categorizing infants into three feeding groups. This is essential as it not only takes out the confounding effects of other infant feeding modes on breastfeeding while studying its association with DNAm, but also allows us to compare the effects of exclusive formula feeding and mixed feedings with recommended exclusive breastfeeding. Also, the use of these feeding modes is consistent with other association studies of infant feeding and adverse health outcomes such as obesity [57], gastroesophageal reflux [58], asthma [59], and food allergy [33]. About 18% of the infants in our cohort were exclusively breastfed for 13 weeks, 8.9% received formula exclusively for 13 weeks, and 73% received formula or solids (along with breastfeeding) before 13 weeks. Mixed feeding constituted the largest group. We used 13 weeks/3 months as a cutoff to categorize each of these three variables to provide sufficient sample size in each group for analysis. 

Our statistical analysis used DNAm differences between birth and age 10 (M values of ΔDNAm or delta residuals) as an outcome by removing the effect of cell-type proportions and season of blood collection at both the time points. Therefore, a positive coefficient between infant feeding and delta methylation implies that DNAm was lower at birth and increased at 10 years following exposure to the infant feeding (EBF/EFF/mixed). On the other hand, a negative coefficient indicates that infant feeding is related to a decrease in DNAm from birth to 10 years. Note, we did not investigate differential DNAm detected in one measurement during childhood, but we did investigate changes of DNAm between birth and age 10 years for different infant feeding modes effective after birth. The use of these delta residual DNAm—compared to a single methylation assessment—is key to interpreting changes in methylation at age 10 with respect to baseline or birth, thus allowing us to examine the real exposure effects of different infant feeding modes on DNAm. For instance, as seen for the mother [60], the maternal wish to breastfeed may already result in a differential methylation at birth, which can be carried forward to childhood DNAm. However, this is not an effect of breastfeeding and would not be falsely detected when investigating changes in DNAm between birth and age 10 years. 

Of the 86 discovered CpGs, we believe that 43 CpGs can be considered as potential biomarkers of infant feeding, since they show a significant effect when comparing DNAm between birth and 10 year and are more stable between 10 and 18 years. 

A potential limitation is that we did not investigate differentially methylated regions (DMRs). There is a possibility that CpGs characterizing DMRs were not discovered by our approach. However, we also have to consider that DMR can be identified using the DNAm profiles in one assessment (at one age) focused either on exposures or diseases, but not for DNAm changes, which we addressed here. For instance, the literature does not suggest that changes in DNAm can be characterized by changes of methylation profiles covering specific genetic regions. In addition, there is a number of limitations involved in the detection and statistical modeling of DMRs [61]. Hence, focusing on CpG dinucleotide as biomarkers more easily facilitates future association studies between infant feeding modes and later health outcomes with CpG dinucleotides as potential mediators. 

Another limitation is that, within the scope of this manuscript, we could not systematically cover the variety of all potential health outcomes that could be related to changes in DNAm. However, we showed potential associations with health outcomes later in childhood for a few continuous health indicators. The selection suggests that changes in DNAm between birth and age 10 may provide novel and interesting insights into later health effects and that changes in DNAm seems to constitute epigenetic biomarker that needs further evaluations. 

Interestingly, infant feeding mode was not only related to specific DNAm but showed an overall wide-spread effect related to a significant decrease in methylation at 292,366 CpGs in exclusively formula-fed infants compared to exclusive breastfeeding. These unspecific consequences are in agreement with a non-specific variety of health outcomes related to infant feeding such as otitis media, lower respiratory tract and gastrointestinal infections, asthma, atopic dermatitis, childhood obesity, type 1 and type 2 diabetes, leukemia, sudden infant death syndrome, and overall infectious morbidity [7,8,9]. 

Although the current study is important for highlighting epigenetic modifications associated with different infant feeding modes, the following factors limit the conclusion of this investigation. Firstly, due to the small sample size in each feeding group and lack of formal replication for generating reproducible results, the findings of the study need to be considered with care. Second, while defining the breastfeeding group, we could not consider other common breastfeeding modes, such as direct feeding at the breast vs. pumping and feeding that have been shown to have different effects on other outcomes studied [35]. Furthermore, despite adjusting for a wide range of confounders in our analysis, there may still be residual confounding that cannot be eliminated due to unknown factors (psychosocial, environmental and nutritional factors related to lactation, etc.), which are not be controlled in the statistical analyses. Lastly, although the study reported reduced DNAm for exclusive formula feeding compared to exclusive breastfeeding, the underlying mechanisms remain unknown. Possible explanations may include either the direct effects of nutritional and bioactive molecules of breastmilk that are absent in formula, or indirectly by the gut microbiota that are impacted by formula, and by differences in the maternal nurturing behavior [62,63]. Also, another study on infant feeding revealed a high expression of *FTO* and *CPT1A* and a low expression of *PPARA* in formula-fed and mixed-fed groups compared to the exclusively breastfed fed group [32]. Hence, whether a decrease in methylation from birth to 10 years observed in case of exclusive formula feeding in the current study is also associated with differential gene expression and different disease risks has yet to be unraveled. 

Future large-scale studies are warranted to evaluate the results of our study. It is crucial to investigate and determine etiologic mechanisms that mediate the observed associations between formula feeding and decreased methylation development up to ten years. Understanding the mechanisms related to this association will first help us to reduce the susceptibility of risks for adverse health outcomes in later life by optimizing infant nutrition. The mechanisms may include bioactive factors in breastmilk or the protective nurturing bond between mother and infant. Second, this information will guide us to develop novel interventions to improve children’s health.

## Figures and Tables

**Table 1:** Comparing characteristics of the analyzed sample consisting of participants with DNA methylation data at both birth and age 10 with the initial Isle of Wight birth cohort.

Variable	Total Cohort (N = 1456)		Participants with DNAm at Birth and Age 10 Year (N = 201)		
	*N*	n (%)	*N*	n (%)	*p*-Value
Gender					
Male	1456	748 (51.3)	201	116 (57.7)	0.07
Maternal smoking					
Yes	1442	362 (25.1)	200	39 (19.5)	0.07
Missing	14		1		
Birth order					
1	1152	491 (42.6)	188	80 (42.6)	0.69
2		394 (34.2)		60 (31.9)	
3		267 (23.2)		48 (25.5)	
Missing	304		13		
Socioeconomic status					
High	1293	104(8)	201	21(10.5)	0.30
Medium		995(76.9)		115(77.1)	
Low		194(15)		25(12.4)	
Missing	163		0		
Caesarean					
Yes	1187	110 (9.3)	167	19 (11.4)	0.36
Missing	269		34		
Maternal asthma					
Yes	1438	158 (10.9)	200	25 (12.5)	0.49
Missing	18		1		
Season of heel prick blood collection at birth					
Winter	1455	417 (28.7)	201	44 (21.9)	0.03
Spring		364 (25)		45 (22.4)	
Summer		352 (24)		61 (30.4)	
Fall		322 (22)		51 (25.4)	
Missing	1		0		
Season of blood collection at 10 years					
Winter	1456	442 (30.4)	201	66 (32.8)	0.23
Spring		285 (19.6)		29 (14.4)	
Summer		225 (15.4)		37 (18.4)	
Fall		504 (34.6)		69 (34.3)	
Birth weight (Kilograms)					
Mean (SD)	1432	3.4 (0.5)	197	3.3 (0.6)	0.19
Missing	24		4		
Maternal age at delivery (years)					
Mean (SD)	1137	29.2 (12.1)	184	29.6 (0.3)	<0.0001
Missing	319		17		
Duration of Breastfeeding (weeks)					
Mean (SD)	1280	14.4 (14.7)	201	15.3 (14.1)	0.35
Missing	176		0		
Age at introduction of formula (weeks)					
Mean (SD)	1304	8.9 (10.9)	201	9.6 (10.5)	0.34
Missing	152		0		
Age at introduction of Solids (weeks)					
Mean (SD)	1252	14.3 (4.6)	201	15.2 (5.2)	0.01
Missing	204		0		
Exclusive breastfeeding (EBF)					
Yes	1319	240 (18.2)	201	46 (22.9)	0.07
Missing	137		0		
Exclusive Formula feeding (EFF)					
Yes	1343	119 (8.9)	201	18 (8.9)	0.98
Missing	113		0		
Mixed Feeding (Breastfeeding/Formula/Solids)					
Yes	1318	956 (72.8)	201	137 (68.2)	0.14
Missing	138		0		

**Table 2:** List of CpGs significantly associated with the different modes of infant feeding.

Feeding Variable	cgID	Coeff ^1^	*p*-Value _Raw_	*p*-Value _FDR_^2^	Gene Name	Chr ^3^	CpG Location	Relation to UCSC ^4^ CpG Island
EBF	cg00367135	0.259	2.04 × 10^−3^	3.90 × 10^−3^	*BEST1*	11	Body	N_Shore
EBF	cg00825951	0.378	1.63 × 10^−4^	1.30 × 10^−3^	*RNF220*	1	5′UTR	Island
EBF	cg00864821	−0.292	4.75 × 10^−4^	1.80 × 10^−3^	*ITIH5; SFMBT2*	10		
EBF	cg01153342	0.207	2.57 × 10^−3^	4.30 × 10^−3^	*C14orf43*	14	TSS1500	S_Shore
EBF	cg01510332	0.204	8.10 × 10^−3^	9.50 × 10^−3^	*MGC2752*	19	Body	Island
EBF	cg01524359	0.245	3.66 × 10^−3^	5.80 × 10^−3^	*NASP*	1	TSS1500	N_Shore
EBF	cg01839850	0.319	3.14 × 10^−4^	1.70 × 10^−3^	*ANKRD54; MIR658*	22	TSS200	Island
EBF	cg02418899	0.246	7.96 × 10^−4^	2.40 × 10^−3^	*ADCYAP1R1*	7	5′UTR	Island
EBF	cg03607728	0.221	1.48 × 10^−3^	3.20 × 10^−3^	*RWDD2B*	21	1stE x on; 5′UTR	Island
EBF	cg05485520	0.237	1.31 × 10^−3^	3.20 × 10^−3^	*LZTFL1*	3	TSS200	Island
EBF	cg05927802	0.275	6.12 × 10^−4^	2.10 × 10^−3^	*ENY2; NUDCD1*	8	Body; TSS1500	Island
EBF	cg07303923	−0.192	5.25 × 10^−3^	7.10 × 10^−3^	*TMEM217*	6	5′UTR	N_Shelf
EBF	cg07399355	0.276	1.52 × 10^−3^	3.20 × 10^−3^	*MUC1*	1	TSS1500	N_Shore
EBF	cg13585032	0.251	4.51 × 10^−4^	1.80 × 10^−3^	*ZNF786*	7	TSS1500	S_Shore
EBF	cg13612447	0.179	1.90 × 10^−2^	1.97 × 10^−2^	*MIR1253*	17		Island
EBF	cg13731777	−0.253	5.13 × 10^−3^	7.10 × 10^−3^	*LGI1; SLC35G1*	10		
EBF	cg14862791	0.208	7.42 × 10^−3^	9.10 × 10^−3^	*TTC23L*	5	5′UTR	Island
EBF	cg15415452	0.410	8.39 × 10^−5^	1.30 × 10^−3^	*SO X 1*	13		Island
EBF	cg16924120	0.188	2.56 × 10^−3^	4.30 × 10^−3^	*SUDS3*	12	TSS1500	Island
EBF	cg17393572	0.215	9.99 × 10^−3^	1.08 × 10^−2^	*HK3*	5	TSS200	
EBF	cg17474435	0.191	9.36 × 10^−3^	1.05 × 10^−2^	*TMBIM6*	12	TSS200	Island
EBF	cg17852997	0.284	1.51 × 10^−3^	3.20 × 10^−3^	*CRYL1*	13	TSS1500	S_Shore
EBF	cg18462168	0.165	3.78 × 10^−2^	3.78 × 10^−2^	*CYFIP1*	15	TSS200	Island
EBF	cg20357023	0.292	1.99 × 10^−4^	1.30 × 10^−3^	*TTC30B*	2	TSS200	Island
EBF	cg23266204	0.209	5.22 × 10^−3^	7.10 × 10^−3^	*EIF2B2*	14	1stE x on	Island
EBF	cg25324653	−0.300	1.11 × 10^−4^	1.30 × 10^−3^	*GHR; FB X O4*	5		
EBF	cg25458520	0.164	6.60 × 10^−3^	8.50 × 10^−3^	*MAPK13*	6	Body	S_Shore
EFF	cg00993400	−0.433	4.04 × 10^−7^	<0.0001	*ZNF692*	1	TSS1500	S_Shore
EFF	cg02351277	−0.364	2.63 × 10^−5^	<0.0001	*TBL1 X R1*	3		
EFF	cg02396836	−0.349	3.53 × 10^−5^	1.00 × 10^−4^	*MIR4255; ZC3H12A*	1		N_Shelf
EFF	cg03144848	−0.364	4.05 × 10^−5^	1.00 × 10^−4^	*GPR20*	8	TSS200	
EFF	cg04017131	−0.449	1.94 × 10^−3^	2.00 × 10^−3^	*ANGPT2* *MCPH1*	8	Body	
EFF	cg04587617	−0.633	3.13 × 10^−6^	<0.0001	*GRB7; IKZF3*	17		
EFF	cg05613803	−0.660	6.97 × 10^−5^	1.00 × 10^−4^	*KIAA0652*	11	Body; 1stE x on; 5′UTR; TSS200	Island
EFF	cg05629774	0.405	3.31 × 10^−5^	1.00 × 10^−4^	*EP400*	12	Body	
EFF	cg05697866	−0.850	9.90 × 10^−6^	<0.0001	*PLCH2; PANK4*	1		S_Shore
EFF	cg07023208	0.340	7.99 × 10^−5^	1.00 × 10^−4^	*TAP2*	6	Body	
EFF	cg07168204	0.491	2.55 × 10^−7^	<0.0001	*NDST1; RPS14*	5		N_Shore
EFF	cg07470634	−0.344	7.15 × 10^−5^	5.00 × 10^−4^	*P X N*	12	5′UTR; Body	
EFF	cg08108289	−0.752	6.97 × 10^−9^	<0.0001	*ZBTB46; ABHD16B*	20		Island
EFF	cg08153335	0.291	4.44 × 10^−4^	5.00 × 10^−4^	*GPR113*	2	5′UTR; 1stE x on; TSS200; Body	
EFF	cg08554115	−0.434	2.76 × 10^−5^	<0.0001	*LOC728392*	17	1stE x on; 3′UTR	Island
EFF	cg08688798	−0.742	1.09 × 10^−5^	<0.0001	*DGCR6L*	22	TSS1500	Island
EFF	cg08704509	−0.757	4.02 × 10^−4^	5.00 × 10^−4^	*GRAMD3*	5	Body; TSS200	N_Shore
EFF	cg09319125	0.377	2.05 × 10^−4^	3.00 × 10^−4^	*RBFO X 3; ENPP7*	17		
EFF	cg09487373	0.399	2.14 × 10^−4^	3.00 × 10^−4^	*KCTD17; TMPRSS6*	22		N_Shelf
EFF	cg10227331	−0.358	4.10 × 10^−4^	5.00 × 10^−4^	*ERRFI1; PARK7*	1		
EFF	cg10309425	0.305	4.63 × 10^−4^	5.00 × 10^−4^	*PRKCI; SKIL*	3		
EFF	cg10382845	0.371	6.12 × 10^−5^	1.00 × 10^−4^	*ABCC11*	16	Body	
EFF	cg11461670	−0.579	1.14 × 10^−6^	<0.0001	*PDLIM2*	8	3′UTR	N_Shore
EFF	cg11597185	0.403	2.81 × 10^−4^	4.00 × 10^−4^	*DHCR7*	11	Body	N_Shelf
EFF	cg12106894	0.388	4.63 × 10^−5^	1.00 × 10^−4^	*KCNK6*	19	Body	S_Shelf
EFF	cg12491659	−0.406	8.05 × 10^−7^	<0.0001	*GAPT*	5	TSS1500	
EFF	cg14027297	0.374	1.61 × 10^−5^	<0.0001	*TFIP11; TPST2*	22		S_Shelf
EFF	cg14161438	−0.509	1.60 × 10^−5^	<0.0001	*ZADH2*	18	Body	Island
EFF	cg14398449	−0.357	5.61 × 10^−5^	1.00 × 10^−4^	*POM121*	7	5′UTR	N_Shore
EFF	cg14737571	0.512	2.90 × 10^−5^	<0.0001	*ATP11A*	13	Body	N_Shelf
EFF	cg15924285	−0.470	9.09 × 10^−5^	2.00 × 10^−4^	*SNORA8; SNORA1* *SNORA32; SNORD6*	11	TSS200; TSS1500	
EFF	cg16746362	0.307	4.03 × 10^−4^	5.00 × 10^−4^	*C6orf25*	6	Body	S_Shore
EFF	cg18555555	0.334	4.36 × 10^−4^	5.00 × 10^−4^	*FABP7*	6	TSS1500	
EFF	cg18990042	−0.258	2.72 × 10^−3^	2.80 × 10^−3^	*COL2A1*	12	TSS1500	Island
EFF	cg19334030	−0.325	8.79 × 10^−4^	1.00 × 10^−3^	*DDAH2*	6	5′UTR	Island
EFF	cg19957727	−0.476	6.08 × 10^−5^	1.00 × 10^−4^	*TBCCD1; DNAJB11*	3	1stE x on; 5′UTR; TSS200	Island
EFF	cg20291674	−0.464	4.97 × 10^−5^	1.00 × 10^−4^	*EZR*	6	5′UTR; TSS1500	Island
EFF	cg22881914	−0.483	2.50 × 10^−4^	4.00 × 10^−4^	*NID2*	14	TSS1500	Island
EFF	cg23528708	−0.377	5.24 × 10^−4^	6.00 × 10^−4^	*CCDC90A*	6	TSS200	Island
EFF	cg23936463	−0.470	6.33 × 10^−6^	<0.0001	*FAM19A5*	22	TSS1500	Island
EFF	cg24456654	−0.414	3.10 × 10^−4^	4.00 × 10^−4^	*PHO X 2B; LIMCH1*	4		S_Shore
EFF	cg24741888	0.278	1.62 × 10^−4^	3.00 × 10^−4^	*USP38; GAB1*	4		
EFF	cg24975769	−0.413	1.05 × 10^−4^	2.00 × 10^−4^	*F X C1; ARFIP2*	11	Body; TSS1500	S_Shore
EFF	cg25350011	−0.526	1.54 × 10^−3^	1.60 × 10^−3^	*ANGPT2;* *MCPH1*	8	Body	
EFF	cg25687894	−0.453	1.06 × 10^−4^	2.00 × 10^−4^	*ACLY*	17	TSS1500	S_Shore
EFF	cg25968437	−0.243	3.06 × 10^−3^	3.00 × 10^−3^	*MLN; LEMD2*	6		
EFF	cg26642844	0.283	5.99 × 10^−4^	7.00 × 10^−4^	*EIF3B*	7	3′UTR	N_Shore
EFF	cg27343836	−0.279	5.23 × 10^−5^	1.00 × 10^−4^	*FO X F1*	16		Island
Mi × ed	cg00809021	0.168	5.85 × 10^−4^	9.00 × 10^−4^	*CISH; MAPKAPK3*	3		
Mi × ed	cg02165095	0.198	2.42 × 10^−3^	2.40 × 10^−3^	*ST8SIA6*	10	Body	
Mi × ed	cg02657836	−0.284	4.33 × 10^−4^	7.00 × 10^−4^	*SGCD*	5		S_Shore
Mi × ed	cg03445694	−0.244	1.34 × 10^−3^	1.50 × 10^−3^	*LH X 8*	1	Body	Island
Mi × ed	cg04086189	0.394	4.44 × 10^−5^	3.00 × 10^−4^	*SLF2; PA X 2*	10		
Mi × ed	cg04797323	0.437	1.31 × 10^−3^	1.50 × 10^−3^	*SOCS2*	12	Body	Island
Mi × ed	cg06491101	−0.311	3.65 × 10^−4^	7.00 × 10^−4^	*RICTOR*	5	Body	
Mi × ed	cg06904618	−0.172	1.63 × 10^−4^	4.00 × 10^−4^	*CT X N3; CT X N3*	5	Body	
Mi × ed	cg08600430	0.266	1.86 × 10^−4^	4.00 × 10^−4^	*SO X 1*	13		N_Shore
Mi × ed	cg09412707	0.246	5.59 × 10^−5^	3.00 × 10^−4^	*SMIM20; SEL1L3*	4		
Mi × ed	cg22976313	0.211	8.91 × 10^−5^	4.00 × 10^−4^	*TMEM179*	14	Body	N_Shore
Mi × ed	cg25324653	0.229	1.05 × 10^−3^	1.40 × 10^−3^	*GHR; FB X O4*	5		

EBF—Exclusively breastfed for at least 13 weeks. EFF—EFF-Exclusively formula fed for at least 13 weeks. Mixed—Given a mixture of breastmilk with formula/solids before 13 weeks. ^1^ Coeff: Regression coefficients represent changes in DNAm from birth to age 10 with each feeding variable. ^2^ FDR: False discovery rate. ^3^ Chr: Chromosome.^4^ UCSC: University of California Santa Cruz.

**Table 3:** Association of infant feeding modes and overall changes in DNA methylation based on 292,366 CpGs from birth to 10 years.

Feeding Mode	Coeff ^1^	Standard Error	*p*-Value
Exclusively formula fed for at least 13 weeks	−0.029	0.012	0.02
Mixture of breastmilk with formula/solids before 13 weeks	−0.004	0.007	0.58
Exclusively breastfed for at least 13 weeks	Reference		

^1^ Coeff: Regression coefficients represent change in overall mean DNAm of 292366 CpGs from birth to age 10 (ΔDNAm residuals controlling for cell types and season of blood collection at both time points) upon exposure to feeding modes (adjusting for confounders).

**Table 4:** Association of the percentages of CpGs that change between birth and age 10 years (delta-residuals) in the lower 25% (below Q1) and in the higher 25% distribution (above Q3) of the methylation of single CpGs related to infant feeding modes.

Feeding Mode	Below Q1 ^1^ (Lowest Quartile)		Above Q3 ^2^ (Highest Quartile)	
	Coeff ^3^ (in Percent)	*p*-Value	Coeff ^4^ (in Percent)	*p*-Value
Exclusively formula fed for at least 13 weeks	4.7	0.001	−0.8	0.57
Mixture of breastmilk with formula/solids before 13 weeks	1.3	0.13	0.4	0.65
Exclusively breastfed for at least 13 weeks	Reference			

^1^ Below Q1 represents percentages of CpGs that are lower than the Q1 level in the children. ^2^ Above Q3 represents percentages of CpGs that are higher than the Q3 level in the children. ^3^ Coeff is the regression coefficient that represents the difference of percentage of CpGs per participant that have delta-residuals below median Q1 cutoff comparing to the reference feeding model after adjusting for confounders (infant’s sex, cell compositions and season of blood collection at birth and age 10 years, maternal age at delivery, maternal smoking during pregnancy, mode of delivery, birthweight, birth order, and family socioeconomic status). ^4^ Coeff is the regression coefficient that represents the difference of percentage of CpGs per participant that has delta-residuals above median Q3 cutoff after adjusting for confounders (infant’s sex, cell compositions and season of blood collection at birth and age 10 years, maternal age at delivery, maternal smoking during pregnancy, mode of delivery, birthweight, birth order, and family socioeconomic status).

**Table 5:** Infant feeding mode and distribution of the CpG in different locations of the gene ^#^.

	Exclusive Breast-Feeding (%)	Exclusive Formula Feeding (%)	Mixed Feeding (%)	Chi-Square Test, *p*-Value
Transcription start sites (TSS)	13 (48)	17 (35)	0	0.04
Not TSS	14 (52)	31 (65)	12 (100)	
Body of the gene incl. Exon 1	5 (18.5)	17 (35.4)	6 (50)	0.009
Not the body of the gene	22 (81.5)	31 (64.6)	6 (50)	

^#^ Whenever multiple locations on the gene were presented (e.g., Body, TSS1500), we counted both locations.

## Data Availability

The data will be available on request from The David Hide Asthma and Allergy Research Centre, St Mary’s Hospital, Isle of Wight PO30 5TG, UK, attention Stephen Potter (ISLE OF WIGHT NHS TRUST) <stephenpotter1@nhs.net>.

## Supplementary Materials

The following are available online at https://www.mdpi.com/2072-6643/13/1/99/s1, Table S1. Overview of previous DNA methylation and breastfeeding studies; Table S2. List of all previously reported CpGs used to investigate overlapping CpGs; Table S3. List of all overlapped genes with prior studies, their corresponding CpGs, and associated feeding variable.
